# E96V Mutation in the *Kdelr3* Gene Is Associated with Type 2 Diabetes Susceptibility in Obese NZO Mice

**DOI:** 10.3390/ijms24010845

**Published:** 2023-01-03

**Authors:** Delsi Altenhofen, Jenny Minh-An Khuong, Tanja Kuhn, Sandra Lebek, Sarah Görigk, Katharina Kaiser, Christian Binsch, Kerstin Griess, Birgit Knebel, Bengt-Frederik Belgardt, Sandra Cames, Samaneh Eickelschulte, Torben Stermann, Axel Rasche, Ralf Herwig, Jürgen Weiss, Heike Vogel, Annette Schürmann, Alexandra Chadt, Hadi Al-Hasani

**Affiliations:** 1 Institute for Clinical Biochemistry and Pathobiochemistry, German Diabetes Center (DDZ), Leibniz Center for Diabetes Research at the Heinrich Heine University Düsseldorf, 40225 Düsseldorf, Germany; 2 German Center for Diabetes Research (DZD e.V.), München-Neuherberg, 85764 Oberschleißheim, Germany; 3 Institute for Vascular and Islet Cell Biology, German Diabetes Center, Leibniz Center for Diabetes Research at Heinrich Heine University, 40225 Düsseldorf, Germany; 4 Max Planck Institute for Molecular Genetics, 14195 Berlin, Germany; 5 Department of Experimental Diabetology, German Institute of Human Nutrition Potsdam-Rehbrücke, 14558 Nuthetal, Germany; 6 Research Group Genetics of Obesity, German Institute of Human Nutrition Potsdam-Rehbrücke, 14558 Nuthetal, Germany; 7 Research Group Molecular and Clinical Life Science of Metabolic Diseases, Faculty of Health Sciences Brandenburg, University of Potsdam, 14469 Potsdam, Germany

**Keywords:** type 2 diabetes susceptibility, pancreatic islet function, insulin secretion, positional cloning, endoplasmic reticulum stress

## Abstract

Type 2 diabetes (T2D) represents a multifactorial metabolic disease with a strong genetic predisposition. Despite elaborate efforts in identifying the genetic variants determining individual susceptibility towards T2D, the majority of genetic factors driving disease development remain poorly understood. With the aim to identify novel T2D risk genes we previously generated an N2 outcross population using the two inbred mouse strains New Zealand obese (NZO) and C3HeB/FeJ (C3H). A linkage study performed in this population led to the identification of the novel T2D-associated quantitative trait locus (QTL) *Nbg15* (NZO blood glucose on chromosome 15, Logarithm of odds (LOD) 6.6). In this study we used a combined approach of positional cloning, gene expression analyses and in silico predictions of DNA polymorphism on gene/protein function to dissect the genetic variants linking *Nbg15* to the development of T2D. Moreover, we have generated congenic strains that associated the distal sublocus of *Nbg15* to mechanisms altering pancreatic beta cell function. In this sublocus, *Cbx6*, *Fam135b* and *Kdelr3* were nominated as potential causative genes associated with the *Nbg15* driven effects. Moreover, a putative mutation in the *Kdelr3* gene from NZO was identified, negatively influencing adaptive responses associated with pancreatic beta cell death and induction of endoplasmic reticulum stress. Importantly, knockdown of *Kdelr3* in cultured Min6 beta cells altered insulin granules maturation and pro-insulin levels, pointing towards a crucial role of this gene in islets function and T2D susceptibility.

## 1. Introduction

Type 2 diabetes (T2D) is one of the most frequently occurring chronic diseases worldwide. Of the 463 million people globally affected by T2D, 72% age between 20–64 years. This number has been continuously rising over the last decades and is predicted to reach 700 million by 2045 [1]. Moreover, taking into account the fact that T2D patients are at increased risk for numerous other afflictions, disease prevention and management has the highest priority. For instance, mortality risk after SARS-CoV-2 infection is 3 times higher in diabetic subjects compared to healthy persons [2]. Individual lifestyle, especially diet and the degree of physical activity, present important risk determinants of T2D onset and progression. In addition, genetic factors constitute a complex element that can also comprise up to 80% of the individual disease susceptibility [3].

Thus, understanding the genetic architecture of T2D has been a hallmark of diabetes research for decades. In the past years, various innovative technologies have been developed that led to an accelerated identification of novel genetic risk factors for T2D [4]. Among these, genome-wide association studies (GWAS) conducted in large populations of unrelated individuals were very successful in identifying common variants associated with human diabetes. However, the genetic variants identified so far account for only a small fraction of the predicted T2D heritability in the human population. Moreover, the causative relationship between most genetic variants and their cellular function with regard to disease susceptibility remain largely unknown [4,5]. The main obstacles in the identification of causal gene variants and their functional role in T2D pathophysiology are the large genetic heterogeneity in the human population and the complexity of an enormous and uncontrolled number of environmental confounding factors [6,7].

In this matter, linkage analysis using selected mouse models has been another relevant approach to identify new genetic risk variants for decades, offering the possibility to substantially reduce genetic heterogeneity by using inbred strains that phenotypically differ in their susceptibility to develop T2D under defined and controlled environmental conditions [8,9]. As one example, the New Zealand obese (NZO) mouse strain is an available mouse model that resembles relevant features of T2D disease in human. Apart from the polygenic arquitecture of T2D development, under high-fat diet-feeding, about 60% of male NZO mice develop the symptoms of the human metabolic syndrome, including morbid obesity, dyslipidemia, vascular complications, insulin resistance, pancreatic beta cells dysfunction and severe hyperglycemia [10]. In contrast, a number of inbred strains, such as the C3HeB/FeJ strain, are known to be resistant towards the development of T2D under equivalent conditions. Previous linkage studies using mouse outcross populations generated with the diabetic-prone NZO versus different diabetes-resistant strains were successful in identifying relevant risk loci for diabetic traits, so-called QTL (Quantitative Trait Loci). Moreover, metabolic characterization of recombinant congenic strains (RCS) generated from intercrossing of diabetes-susceptible with diabetes-resistant mouse strains and thus carrying defined genetic risk loci enable the in vivo investigation of a QTL-dependent impact on disease physiology. In the past, linkage analyses and subsequent positional cloning using the NZO mouse strain have proven to be a successful approach to identify novel risk variants and their respective molecular function [11,12,13]. 

More recently, we and collaborators have conducted a collective crossbreeding approach using the NZO mouse and four different diabetes-resistant breeding partner strains, namely C3H, DBA, B6 and 129P2. Linkage analysis revealed numerous QTL for diabetes-related traits such as blood glucose, body weight and plasma insulin [14,15,16]. Positional cloning studies of the *Nidd/SJL* obesity locus detected in all breeding cohorts of the collective cross project on chromosome 4 yielded mutations in the leptin receptor (*Lpr*) and in the *Zfp69* gene to be causal to this phenotype [17,18]. In addition, a comparative study identified four novel potential genes (*Kti12*, *Osbpl9*, *Ttc39a*, and *Calr4*) associated with pancreatic β-cell loss in the *Nidd/DBA* locus [19]. 

In the crossbreeding of NZO and C3H mice, two novel blood glucose-associated QTL were identified on chromosome 7 and 15. Both loci, named *Nbg7* (NZO blood glucose on chromosome 7) and *Nbg15* (NZO blood glucose on chromosome 15) were associated with a diabetes-protective effect derived from C3H alleles that interact in an additive manner. The genes *Atp4a* and *Pop4* were identified as novel genes associated with *QTL/Nbg7p* [15]. On mouse chromosome 15, previous studies described a QTL linked to diabetogenic traits. However, the specific gene variants underlying this locus and the causal pathophysiological mechanism are not known [20,21,22,23].

Thus, in the present study we performed positional cloning of the *Nbg15* locus by introgressing homozygous C3H alleles onto the NZO genome. Moreover, we went one step further in the identification of the genetic architecture of this locus by addressing gene expression profiling and haplotype analyses combined with advanced bioinformatics models for the impact prediction of DNA variations on gene and protein function. The aim was to unravel the identity and molecular function of the causal *Nbg15* gene variants and address information on possible genes contributing to human T2D susceptibility.

## 2. Results

### 2.1. Heterozygous Carriers of the Nbg15-C3H Alleles Demonstrate Improved Blood Glucose Levels Independently of Their Body Weight

**Figure 1:**
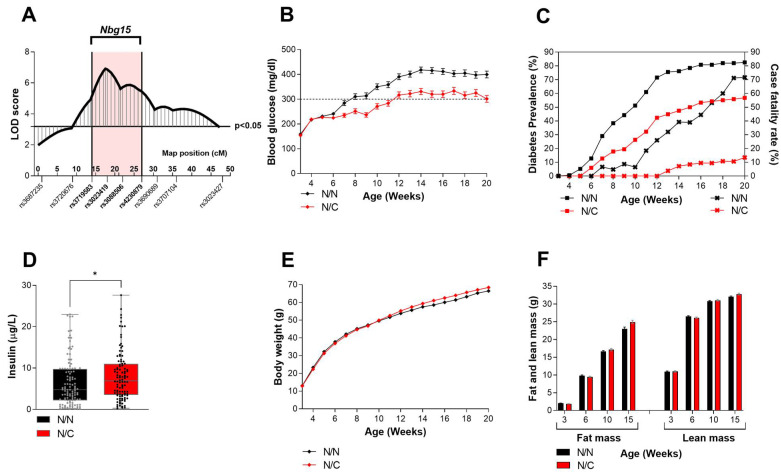
*Nbg15* heterozygous carriers of the C3H alleles demonstrate improved blood glucose levels independently of their body weight. Blood glucose QTL on chromosome 15 identified in the backcross (C3HxNZO)N2 population with the defined *Nbg15* locus (genotyping markers rs37195830 to rs4230879, 50–80 Mbp) (**A**). Allele specific segregation of blood glucose development (**B**), diabetes prevalence (squares) and case fatality rate (x) (**C**), plasma insulin levels (**D**), body weight (**E**), body composition (**F**). LOD scores were calculated using R/qtl package. Allelic segregation was performed using the genotyping markers indicated in bold in the x axis (**A**), *n* = 118–172. Data are presented as box and whiskers (min to max) (**D**) and mean ± SEM (**B**,**E**,**F**) and were analyzed by Students *t* test, * *p* < 0.05 (**D**); or estimated by 100 permutation test in the QTL analysis (**B**,**E**,**F**). Diabetic mice (**C**) were determined as animals with random blood glucose equal or over 300 mg/dL exceeded for at least three weeks in a row. Case fatality rate (**C**) was calculated based on the number diabetic mice that died during the experimental period (20 weeks).

The *Nbg15* locus has previously been identified by our group as a major QTL regulating blood glucose levels in male mice [15]. Here, we aimed to dissect the pathophysiological mechanisms corroborating to this phenotype. An extended set of single nucleotide polymorphism (SNP) markers were used to define the QTL confidence interval. The region on chromosome 15 spanning 50–80 Mbp was defined as the critical *Nbg15* locus, or harboring the main QTL causative genes (Figure 1A). A segregation of the corresponding backcross (NZOxC3H)N2 mice using genotyping markers homogenously distributed over the *Nbg15* locus showed that heterozygous NZO/C3H (N/C) mice displayed substantially lower random blood glucose levels in comparison to homozygous NZO/NZO (N/N) mice (Figure 1B). Furthermore, diabetes prevalence as well as the mortality rate due to diabetes (case fatality rate—CFR) were reduced in the N/C mice compared to the N/N controls (Figure 1C). In addition, plasma insulin levels were significantly higher in the C/N group compared to the N/N mice (Figure 1D). These results, together with the fact that these effects were independent of the body weight or body composition of the mice (Figure 1E,F) led to the assumption that the *Nbg15* from C3H comprises gene variants protecting the pancreatic islets of Langerhans from obesity-induced hyperglycemia. 

### 2.2. Introgression of the Nbg15/C3H Locus into the NZO Genome Protects Mice from Chronic Hyperglycemia

**Figure 2:**
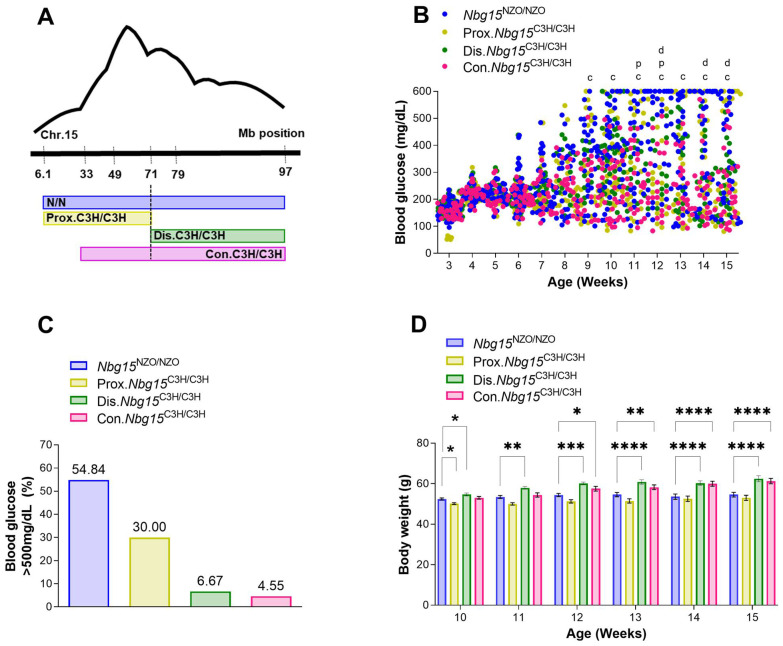
Introgression of the *Nbg15/C3H* locus into the NZO genome protects mice from chronic hyperglycemia. Generation of recombinant congenic lines carrying different genomic fragments of the *Nbg15* locus (**A**). Phenotyping characterization of congenic mice on a high-fat-diet, random blood glucose development (**B**), severe hyperglycemic mice (blood glucose > 500 mg/dL) measured at 15 weeks of age (**C**) and body weight development (**D**). Data are presented as mean ± SEM and were analyzed by two-way ANOVA followed by Bonferroni post hoc-test. * *p* < 0.05; ** *p* < 0.01; *** *p* < 0.001; **** *p* < 0.0001 *n* = 15–45. Letters in B indicate statistical differences comparing the respective recombinant mouse lines (Prox.*Nbg15prox*^C3H/C3H^ = p; Dis.*Nbg15prox*^C3H/C3H^ = d; Con.*Nbg15prox*^C3H/C3H^ = c) carrying the C3H alleles to control group (*Nbg15*^NZO/NZO^). Detailed information on blood glucose and body weight phenotypes are listed in Supplemental Table S1.

To investigate the genetic causality as well as to validate the pathophysiological mechanisms associating the *Nbg15* locus to pancreatic islets integrity, recombinant congenic strains (RCS) were generated carrying either the full *Nbg15*/68.7^C/C^ (consomic, Con.*Nbg15*^C3H/C3H^), proximal *Nbg15*/65^C/C^ (Prox.*Nbg15*^C3H/C3H^*)* or distal *Nbg15*/30.3^C/C^ (Dis.*Nbg15*^C3H/C3H^) *Nbg15* locus from C3H and compared to the control *Nbg15*/95.5^N/N^ group (*Nbg15*^NZO/NZO^) (Figure 2A). Phenotypic characterization of these mice under high-fat diet-feeding (45 cal% from fat) demonstrated a general protection of *Nbg15*/C3H mice from developing hyperglycemia (Figure 2B, Supplemental Table S1). Starting at 9 weeks of age, Con.*Nbg15*^C3H/C3H^ animals displayed significantly lower blood glucose levels in comparison to *Nbg15*^NZO/NZO^ controls. Moreover, blood glucose levels of Prox.*Nbg15*^C3H/C3H^ mice were lower only at weeks 11 and 12. In the Dis.*Nbg15*^C3H/C3H^ group, lower blood glucose levels appeared at later stages of age, reaching statistical significance at weeks 12, 14 and 15 (Figure 2B, Supplemental Table S1). In addition, whereas 55% of *Nbg15*^NZO/NZO^ mice presented blood glucose levels of >500 mg/dL, only 30% of Prox.*Nbg15*^C3H/C3H^ mice displayed severe hyperglycemia. Moreover, only 6% and 5% of the Dis.*Nbg15*^C3H/C3H^ and Con.*Nbg15*^C3H/C3H^ animals, respectively, developed blood glucose levels above this blood glucose threshold (Figure 2C). Body weight was reduced in Prox.*Nbg15*^C3H/C3H^ mice compared to the *Nbg15*^NZO/NZO^ group at weeks 10–13. However, after this period, no statistical differences were observed. The Dis.*Nbg15*^C3H/C3H^ group displayed higher body weight compared to *Nbg15*^NZO/NZO^ after the mice reached 10 weeks of age. After 12 weeks of age also mice from the Con.*Nbg15*^C3H/C3H^ group exhibited higher body weight in comparison to the *Nbg15*^NZO/NZO^ group (Figure 2D, Supplemental Table S2).

### 2.3. Distal but Not Proximal Nbg15 Sublocus Confers Protection from Hyperglycemia in NZO Congenic Mice

**Figure 3:**
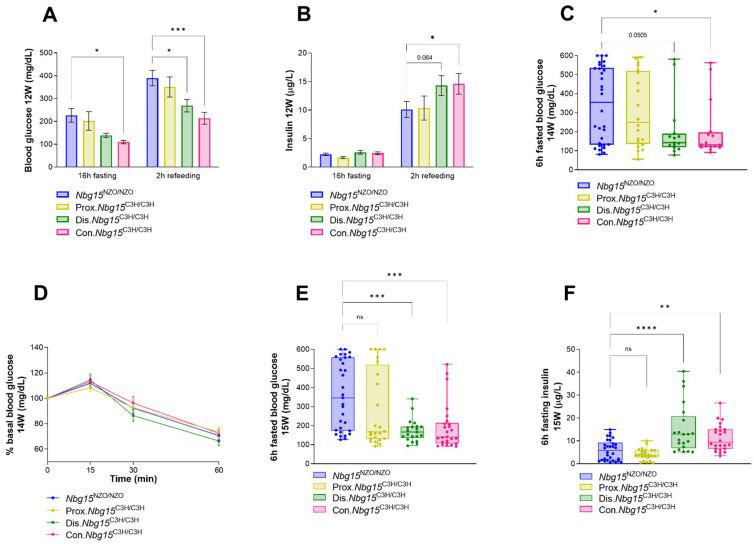
*Nbg15dis^C3H^* but not *Nbg15prox^C3H^* sublocus confers improvements in whole-body glycemia. Mice received high-fat-diet and were subjected to blood glucose (**A**) and plasma insulin (**B**) measurements 16 h after fasting and 2 h after refeeding at week 12 (12 W). 6 h fasted blood glucose prior to insulin tolerance test measured at week 14 (14 W) (**C**), ip.ITT performed at 14 weeks of age (**D**), final 6 h fasted blood glucose at 15 weeks of age (15 W) (**E**) and plasma insulin levels at 15 weeks of age (**F**). Data are presented as mean ± SEM (**A**,**B**,**D**) or min to max. box and whiskers plot (**C**,**E**,**F**) and were analyzed by one or two-way ANOVA followed by Bonferroni post hoc-test, * *p* < 0.05; ** *p* < 0.01; *** *p* <0.001; **** *p* < 0.0001, ns = non significant *n* = 14–31.

In order to elucidate the influence of the two *Nbg15/*C3H subloci on whole-body glycemia, we subjected Con.*Nbg15*^C3H/C3H^, Prox.*Nbg15*^C3H/C3H^, Dis.*Nbg15*^C3H/C3H^ mice and respective *Nbg15*^NZO/NZO^ controls to further metabolic analyses in vivo. In a fasting-refeeding experiment, 16 h-fasted blood glucose levels were significantly lower in the Con.*Nbg15*^C3H/C3H^ group compared to the *Nbg15*^NZO/NZO^ controls. In contrast, the reduction in blood glucose levels was not observed in the Dis.*Nbg15*^C3H/C3H^ or Prox.*Nbg15*^C3H/C3H^ groups when compared to control *Nbg15*^NZO/NZO^ mice (Figure 3A). However, after refeeding, Con.*Nbg15*^C3H/C3H^ and Dis.*Nbg15*^C3H/C3H^, but not Prox.*Nbg15*^C3H/C3H^ mice displayed lower blood glucose levels when compared to the *Nbg15*^NZO/NZO^ control group (Figure 3A). This observation was accompanied by higher plasma insulin levels in Con.*Nbg15*^C3H/C3H^ and Dis.*Nbg15*^C3H/C3H^ mice in the refed state (Figure 3B). Of note, although 6h-fasted blood glucose levels were decreased in Con.*Nbg15*^C3H/C3H^ and Dis.*Nbg15*^C3H/C3H^ animals prior to an intraperitoneal insulin tolerance test, no differences in the response to insulin injection could be observed between the groups (Figure 3C,D). At 15 weeks of age, 6 h-fasted blood glucose levels were significantly lower in Con.*Nbg15*^C3H/C3H^ and Dis.*Nbg15*^C3H/C3H^ but not in Prox.*Nbg15*^C3H/C3H^ mice when compared to the *Nbg15*^NZO/NZO^ controls (Figure 3E). In accordance, plasma insulin levels were increased in Con.*Nbg15*^C3H/C3H^ and Dis.*Nbg15*^C3H/C3H^ compared to control group, but unchanged between Prox.*Nbg15*^C3H/C3H^ and *Nbg15*^NZO/NZO^ animals (Figure 3F).

### 2.4. Distal Nbg15 Sublocus Protects NZO Congenic Mice from Pancreatic Islets Dysfunction

**Figure 4:**
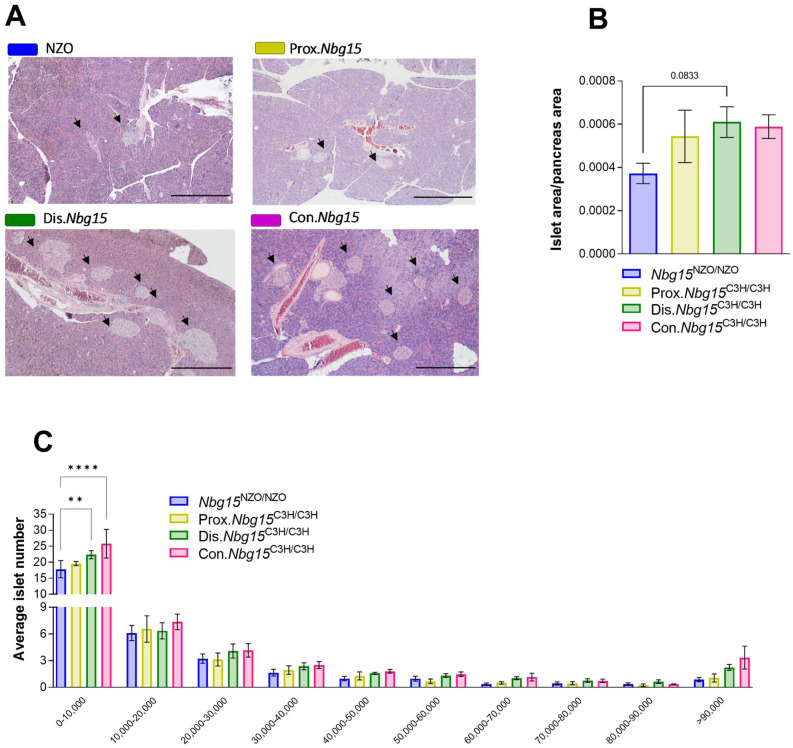
*Nbg15dis^C3H^* but not *Nbg15prox^C3H^* sublocus protects NZO mice from pancreatic islets dysfunction. Representative section for islets size distribution within the genotypes on a high-fat-diet, islets in each section are indicated with arrows (**A**), islet area/ pancreas area (**B**), islet size distribution (**C**). Data are presented as mean ± SEM and were analyzed by one or two-way ANOVA followed by Bonferroni post hoc-test, ** *p* < 0.01; **** *p* < 0.0001 *n* = 3–7.

To investigate morphological differences in pancreatic islets of Langerhans, whole pancreata from fasted Con.*Nbg15*^C3H/C3H^, Prox.*Nbg15*^C3H/C3H^ and Dis.*Nbg15*^C3H/C3H^ mice and respective *Nbg15*^NZO/NZO^ controls were subjected to histomorphometrical analyses via HE (hematoxylin/eosin) staining (Figure 4A). Islet area/pancreas area was slightly increased in the Dis.*Nbg15*^C3H/C3H^ group in comparison to *Nbg15*^NZO/NZO^ mice (Figure 4B), whereas no differences in the average pancreatic area was observed for this comparison (Supplemental Figure S1). Moreover, the majority of counted islets were assigned to a cluster of smaller islets (0–10,000 mm^2^). Within this cluster, both the Con.*Nbg15*^C3H/C3H^ and Dis.*Nbg15*^C3H/C3H^ genotypes had a higher number of pancreatic islets when compared to the *Nbg15*^NZO/NZO^ controls, whereas no changes were observed in sections from Prox.*Nbg15*^C3H/C3H^ mice (Figure 4C).

### 2.5. Kdelr3 Represents a Potentially Causal Gene for the Diabetes-Protective Distal Sublocus

**Figure 5:**
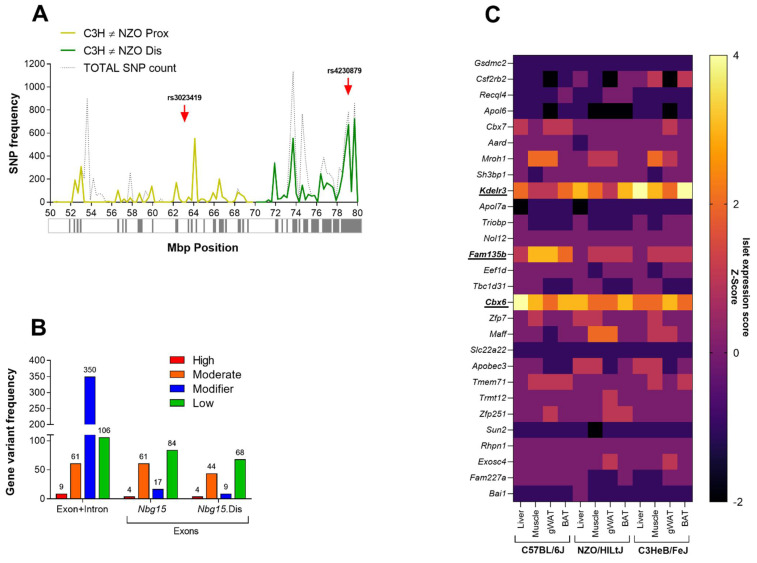
Combined approaches successfully identified potential causative genes underlying the *Nbg15dis* sublocus. Identification of C3H polymorphic regions underlying the *Nbg15* sub-loci (**A**), variant effect prediction analysis (VEP) of identified non-IBD SNPs (**B**) and heatmap of islet specific genes containing non-IBD SNPs predicted to affect protein effectiveness selected via the VEP analysis and the SIFT code under 0.5. Polymorphic regions were calculated using Genome Reference Consortium Mouse Build 38 provided by the Wellcome Trust Sanger Institute and defined as more than 50 SNPs/150 Kb window (gray boxes in (**A**)). Red arrows (**A**) indicate the QTL peak markers of each *Nbg15* sub-loci. VEP analysis is a free available tools from Ensembl and uses SnpEff (Open Source tool) tool to calculate subjective changes in proteins containing the SNPs or amino acid exchanges. SIFT (Sorting Intolerant from Tolerant) is an additional VEP score that consider physical properties of amino acid exchanges to determine its protein impact. Microarray expression of mouse parental VEP and SIFT sorted genes were used to generate the heatmap for pancreatic islet specific expressed genes. The ratio of islet expression vs. liver, muscle (quadriceps), gWAT (gonadal white adipose tissue) and BAT (brown adipose tissue) was calculated as Z-Score for the NZO, C3H and B6 mouse strains. Genes with high scores observed for all mouse strains and tissues were considered as main candidates for the *Nbg15* locus (**C**).

**Figure 6:**
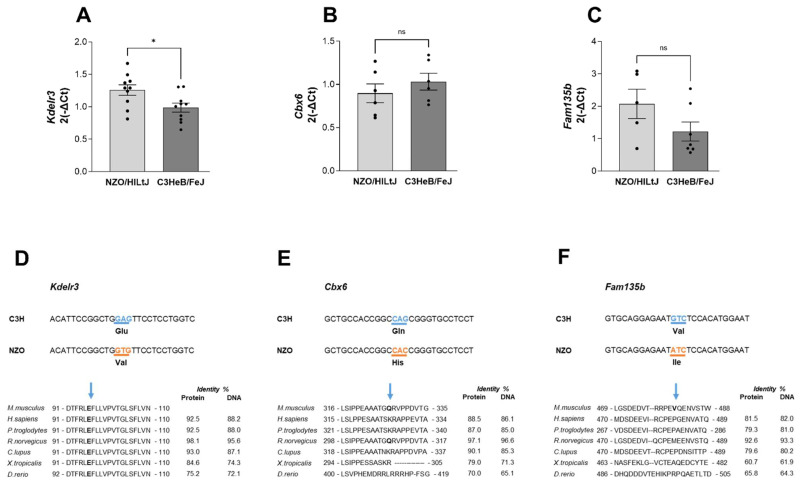
Gene expression profiling and multiple species amino acid sequence alignment of *Nbg15 nominated genes* in the pancreatic islets. Quantitative real-time PCR of *Kdelr3* (**A**), *Cbx6* (**B**) and *Fam135b* (**C**) in isolated pancreatic islets of parental C3H and NZO mice. Validation of identified polymorphic SNPs within *Nbg15* locus and multiple species alignment of the respective protein containing the missense mutation (**D**–**F**). Data are presented as mean ± SEM and were analyzed by Students *t* test, * *p* < 0.05; ns = non significant *n* = 5–10.

The phenotypic characterization of the RCS mouse lines harboring different fragments of the *Nbg15* locus (*Nbg15con, Nbg15prox, Nbg15dis)* indicated the *Nbg15dis* sublocus to contain the causative gene variants protecting pancreatic islet cells from obesity-related chronic hyperglycemia. Next, haplotype analysis was conducted to determine informative SNPs that differ between the C3H and NZO mouse strains. The *Nbg15dis* sublocus featured a higher number of polymorphic SNPs compared to the *Nbg15prox* sublocus (8435 vs. 5084 SNPs) (Figure 5A). Further, the identification number for each SNP was used to predict non-synonymous exchanges and to score out the genes that have less potential to drive deleterious effects (Figure 5B). In total, 66 genes were identified in the *Nbg15* locus containing SNPs predicted to have a moderate impact on protein level, the majority (44) also located in the distal sublocus. Moreover, 4 genes were identified featuring SNPs with a high prediction to affect protein function, all located in the *Nbg15dis* sublocus. Subsequently, genes classified in the so-called “moderate group” were scored according to the SIFT (Sorting Intolerant from Tolerant) code of deleteriousness. Thus, 30 genes with a SIFT code lower than 0.5 were further considered in the analysis. Since the phenotypic characterization of the congenic lines demonstrated that C3H alleles protect NZO mice from pancreatic islets dysfunction, we specifically focused on genes expressed in pancreatic islets of Langerhans. For this purpose, microarray analysis performed in different tissues of the parental C3H, NZO and B6 mice was conducted to screen for genes predominantly expressed in the pancreatic islets when compared to other diabetes-driven target tissues (Figure 5C, Supplemental Table S3). The results led to the identification of *Kdelr3, Cbx6* and *Fam135b* as genes highly expressed in pancreatic islets of Langerhans and potentially causative genes underlying the *Nbg15* locus. Next, expression levels of the three candidate genes *Kdelr3, Cbx6* and *Fam135b* were assayed via quantitative real-time PCR (RT-qPCR) in isolated pancreatic islets of Langerhans from the two parental strains NZO and C3H. Expression levels of *Kdelr3* were significantly higher in NZO islets compared to C3H islets, while no differences were observed for the genes *Cbx6* and *Fam135b* (Figure 6A–C, Supplemental Figure S2A). We performed a multiple species sequence alignment that detected a missense mutation in the *Kdelr3* (E96V) gene located in a highly conserved protein region (Figure 6D), while the detected mutations in the *Cbx6* and *Fam135b* (Q309H, V485I) genes were conserved exclusively in rodents (Figure 6E,F).

### 2.6. Changes in Kdelr3 Gene Expression Lead to Alterations in Insulin Granule Maturation and Upregulation of ER Stress Signals in Pancreatic Beta Cells

**Figure 7:**
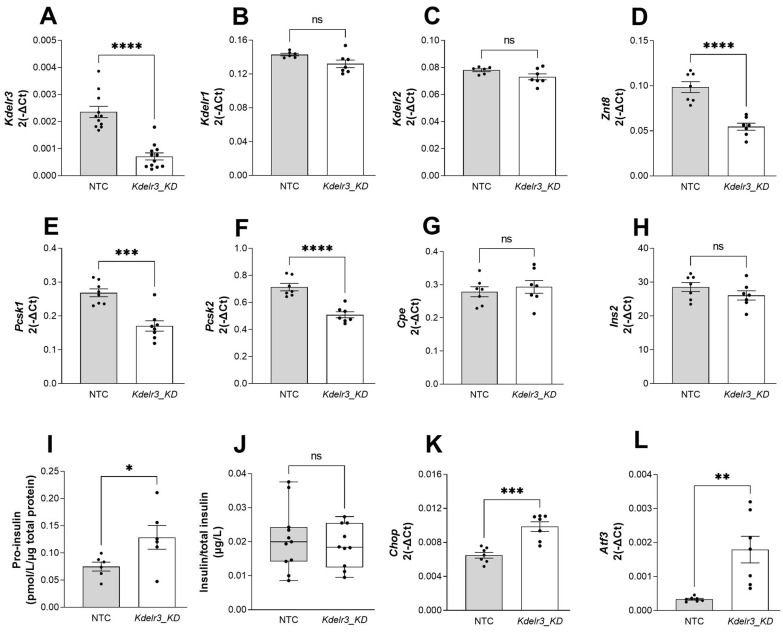
Effect of *Kdelr3* Knockdown in MIN6 insulinoma cells. Quantitative real-time PCR of *Kdelr*3 (**A**), *Kdelr1* (**B**), *Kdelr2* (**C**), *Znt8* (**D**), *Pcsk1* (**E**), *Pcsk2* (**F**), Cpe (**G**) and *In2* (**H**). Pro-insulin in measured in MIN6 cell lysates (**I**), GSIS after 25 mM glucose treatment (**J**). Quantitative real-time PCR of *Chop* (**K**) and *Atf3* (**L**). *Kdelr3* knockdown cells were generated using a pool of siRNA. Data are presented as mean ± SEM and were analyzed by Students *t* test, * *p* < 0.05; ** *p* < 0.01; *** *p* <0.001; **** *p* < 0.0001; ns = non significant *n* = 5–12 independent experiments.

Following the identification of *Kdelr3* as a major candidate gene underlying the *Nbg15* locus, we further focused on the functional in vitro characterization of the *Kdelr3* gene. RNA interference-mediated knockdown (KD) of *Kdelr3* in the insulinoma cell line MIN6 led to a significant reduction (70%) of *Kdelr3* gene expression compared to cells transfected with the non-target control siRNA oligonucleotides (NTC) (Figure 7A). Furthermore, no compensatory alterations in the expression levels of related *Kdel* receptor genes were observed upon *Kdelr3* KD (Figure 7B,C). As a result of the *Kdelr3* depletion in MIN6 cells, the genes for zinc transporter 8 (*Znt8*) and Proprotein Convertase Subtilisin/Kexin Type 1 and 2 (*Pcsk1, Pcsk2*) were significantly downregulated (Figure 7D–F) with no alteration in the carboxypeptidase E (*Cpe*) gene (Figure 7G). Notably, while mRNA levels of insulin 2 (*Ins2*) were not affected by the *Kdelr3* KD, protein levels of pro-insulin were significantly higher in lysates of the *Kdelr3* KD cells in comparison to the NTC (Figure 7H,I). Moreover, no changes in glucose-stimulated insulin secretion or total insulin content were observed upon downregulation of *Kdelr3* (Figure 7J, Supplemental Figure S3A). In addition, gene expression levels of the two ER-stress markers C/EBP homologous protein (*Chop*) and Activating Transcription Factor 3 (*Atf3*) were significantly higher after *Kdelr3* KD compared to NTC cells (Figure 7K,L). Moreover, the markers for beta cell identity MAF BZIP Transcription Factor A (*Mafa*) and pancreatic and duodenal homeobox 1 (*Pdx1*) were unaltered by the knockdown of *Kdelr3* in MIN6 cells. In addition, the marker of proliferation (*Ki67*) was downregulated in knockdown cells compared to control group (Supplemental Figure S3B–D).

## 3. Discussion

A linkage study using a NZOxC3H crossbreeding approach was previously performed in our group and led to the identification of *Nbg15* as a major QTL regulating blood glucose levels on mouse chromosome 15 [15]. T2D-associated phenotypes linked to chromosome 15 have been described in a number of linkage studies from other groups, namely in the insulin deficient (AkitaxA/J)F2 mice, carriers of diabetes mutation (BKSxDBA/2)F2 mice, (A/WySnJxCAST/Ei) mice and (NZO/HlLtxNON/Lt) animals [20,24,25,26]. Nevertheless, the respective causal gene variants and in particular the molecular function underlying these loci remain to be identified. For this purpose, we combined mouse positional cloning of the *Nbg15* locus with advanced bioinformatics analysis of DNA variations to identify potential causal candidate genes linked to the *Nbg15* locus.

In the present study, a fine mapping of the *Nbg15* locus revealed two defined subloci at 63 Mbp (proximal) and 79 Mbp (distal), respectively. When combined, both subloci were associated with differences in blood glucose levels that reached an average of 93 mg/dL. The diabetes-protective effect of the *Nbg15* C3H alleles was not attributed to alterations in body weight or body composition. Moreover, *Nbg15* C3H allele carriers display substantially elevated plasma insulin levels, leading to the hypothesis that *Nbg15* may primarily act by altering the function of islets of Langerhans and not by influencing insulin sensitivity in peripheral tissues.

To confirm this hypothesis and investigate the phenotypic contribution of each *Nbg15* sublocus to the phenotype, we generated RCS lines carrying the respective *Nbg15* subloci from C3H into the NZO genome. The results demonstrated that protection from hyperglycemia mainly derives from C3H genes located in the distal part of the *Nbg15* locus. Moreover, improved glycemic control was associated with a higher insulin secretion and increased number of islets in these mice, driving the nomination of *Nbg15* causal genes to the distal sublocus. This evidence, together with the observation of loss of body weight due to diabetes and lack of plasma insulin in the control NZO group compared to the distal carriers of the C3H alleles, strongly supports the notion that genes in this locus act almost exclusively protecting beta pancreatic cells from dysfunction. Of note, a contribution of the proximal *Nbg15* sublocus in diabetes protection cannot be excluded due to the observed overall decreased in hyperglycemia incidence and transient ameliorated random blood glucose levels. 

We went further in the investigation of the *Nbg15*-dependent diabetes-protection by analyzing DNA variations in the parental C3H and NZO mouse strains in order to improve the mapping of causative genes within the QTL [27,28]. Genes containing non-identical by descendent SNPs (non-IBD SNPs) were assumed to be more likely to cause the observed phenotype. In this study, we also integrated in silico predictions of gene and protein functional impact of each polymorphic SNPs annotated in the *Nbg15* locus. Interestingly, the genomic region corresponding to the distal sublocus of *Nbg15* was shown to harbor the majority of the polymorphic blocks and genes containing SNPs predicted to affect protein function, once more supporting the hypothesis of the distal *Nbg15* sublocus as predominant facilitator of diabetes protection in this region. Furthermore, microarray gene expression profiling was used to filter for genes specifically expressed in pancreatic islets of Langerhans. The results led to the identification of *Kdelr3* (endoplasmic reticulum protein retention receptor 3), *Cbx6* (chromobox 6) and *Fam135b* (family with sequence similarity 135, member B) as potential candidate genes underlying the *Nbg15* locus that possibly regulate pancreatic beta cell function. The Tabula Muris database for single-cell transcriptomic data of 20 mouse organs sustained the finding of the genes *Kdelr3*, *Cbx6* and *Fam135b* being highly expressed in pancreatic islets cells [29]. Interestingly, all amino acid exchanges in these candidate genes were exclusively found in the NZO genome. In the CBX6 protein, we detected an amino acid exchange where the uncharged glutamine is exchanged to the positively charged histidine. In the FAM135B protein, an exchange of valine to isoleucine was found. However, according to our analyses, these variations were classified as better tolerated using the SIFT code tool. In contrast, in the KDELR3 we detected an exchange of the negatively charged glutamic acid to the uncharged valine (Glu^96^). This mutation was classified as having a higher impact in protein function (SIFT 0.08), due to the nature of physical property exchanges as well as the location of the mutation in a highly conserved protein region among species. In addition, RT-qPCR analysis of pancreatic islets isolated from parental C3H and NZO mice that were subjected to a high-fat diet for 6 weeks identified no differences in the expression of *Cbx6* and *Fam135b*. However, the gene expression levels of *Kdelr3* were significantly higher in NZO islets compared to the C3H mice. KDELR3 is the third member of this protein family that is known to control the retention or transport of proteins from the ER to Golgi via interaction with specific C-terminal amino acids motifs. In addition to a high degree of species conservation, the three members of this family share 73–83.5% pairwise amino acid identity. According to a recent study on the structure of KDELR2, members of this receptor family are organized in 7 transmembrane (TM) alpha helices and two internal triple helix bundles (THB), connected by the linker helix TM4. Upon pH acidification, conformational changes occur to form a negatively charged pocket for ligand binding [30]. The mutated residue at position 96 (Glu^96^) identified in the NZO mouse strain is located in the TM4. This region was described to be relevant for protein function in studies with evolutionary related SWEET transporters [30,31]. In addition, it has been postulated that KDEL receptors present specific affinities for ligand binding and interact with different KDEL motifs, that may require different protein interaction sides yet to be identified [32]. Moreover, the fact that humans express three different KDELR3 isoforms was suggestive of a participation of these receptors in multiple cellular functions [33,34]. In our findings, from all three *Kdel* genes, only the mRNA levels of *Kdelr3* were altered in the pancreatic islets of NZO compared to C3H, corroborating with possible specialized function of specific *Kdel* members. Recently, the participation of KDELR3 in the unfolded protein response (UPR) has been described [34]. Moreover, the KDEL family of proteins was also suggested to participate in the intracellular signaling mechanisms that promote the trafficking of proteins from the Golgi to the plasma membrane via interaction with proto-oncogene tyrosine-protein kinase Src proteins, protein kinase A (PKA) and protein kinase C (PKC) [33,35]. These processes have been implicated to directly affect the biosynthesis, maturation and secretion of insulin granules in beta cells [36]. Thus, we further investigated the impact of a *Kdelr3* depletion in the insulinoma beta cell line MIN6. We observed a downregulation of *Znt8*, *Pcsk1* and *Pcsk2* but not *Cpe* genes and higher levels of pro-insulin in knockdown cells, linking *Kdelr3* function to insulin granule maturation, presumably influencing beta cell functionality. Although no significant changes were observed in glucose-stimulated insulin secretion and total insulin content upon *Kdelr3* knockdown, the elevated pro-insulin content in knockdown cells points towards an influence of KDELR3 in insulin processing and beta cells function. For instance, increased pro-insulin levels have been described as an early indication of beta cell loss in human type 1 diabetes [37,38]. In addition, the ER stress markers *Chop* and *Atf3* were upregulated in *Kdelr3* knockdown cells, presumably also affecting cell cycle arrest resulting in the downregulation of *Ki67* [39]. In a previous study, *Kdelr3* upregulation was observed upon treatment of the insulinoma INS1-E cells with the ER stress-inducing compound Thapsigargin. Our study also detected increased *Kdelr3* expression when isolated pancreatic islets from NZO mice were subjected to an overnight cellular stress intervention induced by glucolipotoxicity. In addition, available data of gene expression in human pancreatic islets show a strong positive correlation of *KDELR3* with insulin expression [40]. Moreover, elevated *KDELR3* expression was recently reported in patients with hepatocellular carcinoma and associated with other genes as markers for a negative disease prognosis [41]. Importantly, Wang et al. (2021) found mutations in the *KDELR3* gene in a fraction of the patients affected by hepatocellular carcinoma, all of these still with so-far unknown significance. Consequently, the possibility that the upregulation of *Kdelr3* mRNA may also result from compensatory mechanisms associated with the existence of a missense variant must be taken into account. Moreover, proteins involved in protein maturation such as chaperones and isomerases contain KDEL retention motifs. This is another evidence that links *Kdelr3* to the susceptibility of T2D diabetes, given the reports of overloaded misfolded proteins as hallmark for beta cell dysfunction and apoptosis in the disease. However, this specific function has not been investigated in this study [42,43,44]. Finally, malfunction in electrostatic interaction of the NZO KDELR3 mutant with proteins carrying the KDEL motif could result in gene upregulation and consequently alter signals for ER stress response, ultimately leading to cell death and mark the initiation of pancreatic beta cell dysfunction associated with T2D onset. 

### Conclusion and Future Perspectives

The genetic basis of T2D susceptibility in human remains poorly understood. Mouse linkage studies represent a powerful tool in the identification of disease-associated genes. For this purpose, our group has generated a mouse outcross population between the diabetes-prone NZO and diabetes-resistant C3H mouse strains. Linkage analysis identified *Nbg15* as a novel locus associated with T2D susceptibility. The present study aimed to unravel the causal genes and the pathophysiological mechanism underlying to the *Nbg15* locus. Introgression of the *Nbg15* genomic fragments from the C3H strain into the NZO genome demonstrated that diabetes protection in the *Nbg15* carriers of the C3H alleles resulted from genes located distally in this locus. Moreover, potential identified mechanisms associated with *Nbg15* involve the maturation of insulin granules and/or pancreatic beta cell survival. A combination of gene expression profiling and analysis of DNA variations within the distal sublocus of *Nbg15* identified *Cbx6*, *Fam135b* and *Kdelr3* as genes potentially linked to the observed phenotype. Nonetheless, the identification of a *Kdelr3* mutation in the NZO genome in a highly conserved domain as well as alterations on the mRNA levels of this gene in pancreatic islets of Langerhans favored this gene as most likely candidate influencing beta cell function in our model. We hypothesized that the identified mutation may alter electrostatic interactions of the KDEL motif in the beta cell and thus negatively influence adaptive responses required for the management of ER-overloaded protein scenario in a pre-diabetic or diabetic beta cell. However, the exact mechanism underlying the association of *Kdelr3* to diabetes risk and to beta cell stability remains to be clarified in future studies.

## 4. Materials and Methods

### 4.1. Chemicals and Buffer

Chemicals and buffer ingredients are listed in Supplemental Table S4.

### 4.2. Experimental Animals

All animal experiments were approved by the Ethics Committee of the State Ministry of Agriculture, Nutrition and Forestry (State of North Rhine-Westphalia, 45659 Recklinghausen, Germany) (references: 84-02.04.2013.A118, 84-02.04.2015.A354 and 81-02.04.2021.A168). Three to six male (3–20 weeks old) mice per cage were housed at 22 °C and a 12 h light–dark cycle (lights on at 6 a.m.) with ad libitum access to food and water. After weaning, the mice were fed a high fat diet containing 45 kcal% fat, 20 kcal% protein, and 35 kcal% carbohydrates with 4.73 kcal/gm energy (D12451; Research Diets, New Brunswick, NJ, Canada). The generation of the backcross population and recombinant congenic strains was performed as previously described [15]. Congenic strains of generations N_5_F_2-5_/N_6_F_2-5_ underwent a metabolic characterization under high fat diet intervention until 15 weeks of age. At this age the mice were fasted for 6 h and killed by decapitation. The mice were continuously genotype using the SNPs panel presented in the Supplemental Table S5. Genotyping was performed using Kompetitive Allele-Specific PCR from LGC genomics (LGC group, Teddington, UK).

### 4.3. Blood Glucose, Body Weight and Body Composition

Blood glucose was assessed weekly by pricking tail blood and measuring with a glucometer (Contour, Bayer Consumer Care AG, Leverkusen, Germany). Body weight was measured weekly with an electronic scale, and body composition assessed by non-invasive nuclear magnetic resonance spectroscopy at 3, 6, 10 and 15 weeks of age (EchoMRI-100 System; Echo Medical Systems, Houston, TX, USA).

### 4.4. Fasting/Refeeding and Insulin Tolerance Test

For fasting and refeeding experiments tail blood was collected after fasting the mice overnight for 16 h and after 2 h of refeeding. The blood was centrifuged at 13,000 rpm at 4 °C and plasma collected for the insulin measurements. For the insulin tolerance test mice were fasted in the morning for 6 h. After that, insulin (Actrapid, Novo Nordisk, Bagsværd, Denmark) 1 IU/Kg body weight was injected intraperitoneally. The drop in blood glucose was monitored at 0, 15, 30 and 60 min after the injection. The blood glucose measurements were obtained using a glucometer.

### 4.5. Plasma Insulin

Insulin was measured in plasma samples using ELISA kit (Insulin: Mouse Ultrasensitive ELISA Kit; DRG, Marburg, Germany). Fasting and refeeding blood was collected at 12 weeks of age in a microvette coated with Lithium-Heparin (CB 300, Sarstedt, Germany). At 15 weeks of age, trunk blood was collected in a micro tube coated with an anticoagulant cocktail as indicated in Supplemental Table S4.

### 4.6. Pancreas Histology and Islets Isolation

Dissected pancreas of 15 weeks old mice was immediately fixed with 4% paraformaldehyde for 24 h and embedded in paraffin after dehydration. Thereafter, a series of 15 consecutive 5 µm sections were selected every 75 µm of the tissue block. The obtained sections were stained with hematoxylin/eosin. Total pancreas area was determined by point counting as previously described [45]. The determination of the area was calculated by using the Wacom Tablet and the polygon tool of the CellSens Dimension 1.9 software (Olympus) after manually surrounding the perimeters of each islet. Islets of parental mice were isolated as previously described [45].

### 4.7. Cell Culture and Knockdown Experiments

MIN6 cells (a gift from S. Baltrusch from the University of Rostock, Germany) were cultivated at 37 °C with 5% CO_2_ in Dulbecco’s Modified Eagle Medium (Thermo Fisher Scientific) with 25 mM glucose, supplemented with 10% fetal calf serum and 1% penicillin/streptomycin. For knockdown experiments, 0.5 M cells were seeded in a 6 well and cultured overnight before they were transfected with 50 µM of a pool of non-target (NT) siRNA or *Kdelr3* siRNA (Dharmacon, Horizon Discovery Biosciences Ltd., Cambridge, UK) (Supplemental Table S6). Transfections were performed using Lipofectamine 2000 (Thermo Fisher Scientific, Waltham, MS, USA) as vehicle according to the manufacturer’s instructions (Thermo Fisher Scientific). 48 h hours after transfection the cells were washed with PBS and collected in trizol for further RNA isolation. For the glucose stimulated insulin secretion (GSIS), *Kdelr3* knockdown cells and controls were subjected to 1 h pre-incubation with Krebs-Ringer HEPES (KRH) buffer. Subsequently, the cells were incubated for 1 h with KRH supplemented with 25 mM glucose. The supernatant and cell lysates were collected for the measurement of secreted and total insulin or pro-insulin content using ELISA Kits (Insulin: Mouse Ultrasensitive ELISA Kit; DRG, Marburg, Germany; Pro-insulin: Mercodia AB, Uppsala, Sweden) (Supplemental Table S4). Lysates protein content was determined by bicinchoninic acid (BCA) assay (Pierce Chemical, Rockford, IL, USA).

### 4.8. RNA Extraction, cDNA Synthesis and Quantitative Real-Time PCR

RNA was extracted using QIAzol Lysis Reagent (QIAGEN, Hilden, Germany) and cDNA synthesis performed with GoScript™ Reverse Transcriptase system (Promega, Madison, WI, USA) according to manufacture’s instruction. Self-designed Sybr-Green PCR primers (Supplemental Table S7) were used for the determination of gene expression by quantitative real-time PCR. Gene expression in MIN6 cells were calculated with ΔCt method and actin beta (*Actb*) used as housekeeping gene [46].

### 4.9. Sequence, Haplotype and Gene Variants Analysis

Information on mouse SNPs was obtained from the Sanger Wellcome Trust Institute Database (https://www.sanger.ac.uk/sanger/Mouse_SnpViewer, accessed on 20 November 2022) [47,48,49]. Prediction of functional impact of SNPs in the corresponding genes and proteins were made using the Ensembl Variant Effect Predictor (VEP—https://www.ensembl.org/Tools/VEP, accessed on 20 November 2022) and the algorithm “Sorting Tolerant From Intolerant” (SIFT—http://sift.jcvi.org, accessed on 20 November 2022) [50,51,52]. Determination of polymorphic region were defined by a frequency of more than 50 non-identical by descent SNPs in an interval of 150 Kbp. Annotated SNPs for the Mouse Build 38 (C57BL/6J) were used as reference and counted for the C3H/HeJ and NZO/HlLtJ strains.

As described before, intervals of 250 kbp were selected for the determination of the frequency of polymorphic SNPs between the different mouse strains, and a threshold of 100 SNPs /window was chosen to distinguish the QTL into regions that are identical-by-descent (IBD, genomic regions that are identical between individuals due to descent from a common ancestor without recombination) and polymorphic (non-IBDs) between the different mouse strains. For the determination of the total number, all SNPs annotated for the C57BL/6J reference genome with calls for C3H/HeJ, 129P2/OlaHsd, and NZO/HlLtJ were counted [15,53].

### 4.10. Statistics

Unless otherwise stated, data are reported as mean ± SEM. Significant differences were determined by one-way or two-way ANOVA (post hoc test, Bonferoni correction) or unpaired, two-tailed Students *t* test, as indicated in Figure legends. Quantitative trait loci analysis were performed as previously described [15]. The confidence interval for the *Nbg15* locus was determined using the 1.5 LOD interval and the nearest genotyping marker. *p* values < 0.05 were considered statistically significant. Microarray data are available under accession number GSE117553 and were analyzed as previously described [15].

## Data Availability

The data presented in this study are available on request from the corresponding author.

## Supplementary Materials

The following supporting information can be downloaded at: https://www.mdpi.com/article/10.3390/ijms24010845/s1.
