# Supplementary material for: E96V Mutation in the Kdelr3 Gene Is Associated with Type 2 Diabetes Susceptibility in Obese NZO Mice
Source: Int J Mol Sci. 2023 Jan 3;24(1):845. doi: 10.3390/ijms24010845 (PMC9820861; doi:10.3390/ijms24010845)
Supplement: Supplementary file 1 [file ijms-24-00845-s001.zip › ijms-2097810-supplementary.pdf]

International Journal of
Molecular Sciences

MDPI

Supplemental Material

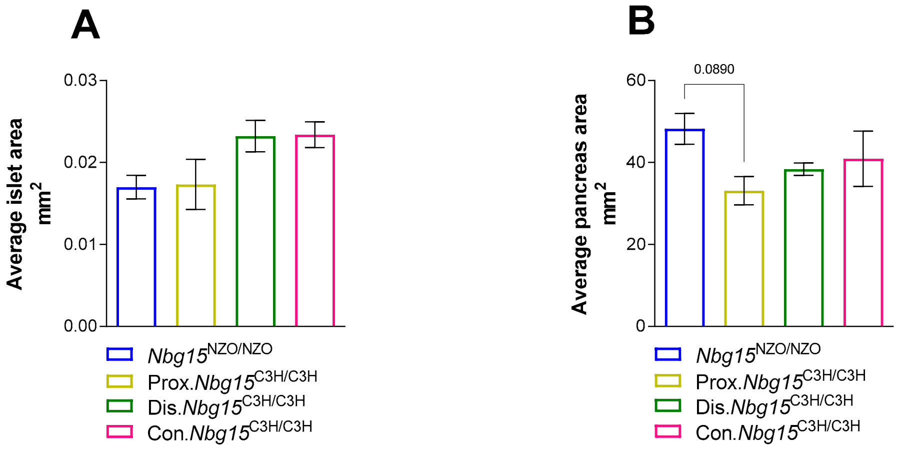

Supplemental Figure S1. Pancreata histological analysis of congenic strains for the *Nbg15* locus. Average islet area (A) and average pancreas area (B). Data are presented as mean ± SEM and were analyzed by one ANOVA followed by Bonferroni post-hoc-test, *p < 0.05; **p < 0.01; ****p < 0.0001 n=3-7.

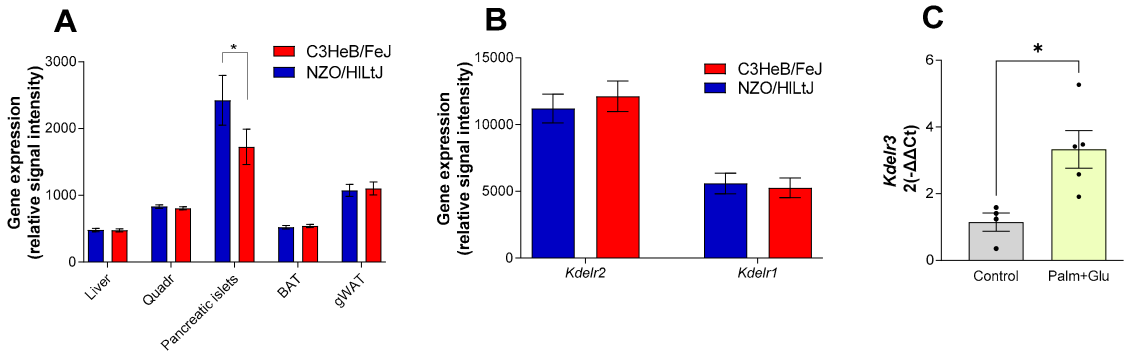

Supplemental Figure S2. Microarray gene expression profiling of *Kdelr3* in liver, muscle (quadriceps), pancreatic islets, brown adipose tissue (BAT) and gonadal adipose tissue (gWAT) in parental C3H and NZO mice (A). Expression levels of *Kdelr2* and *Kdelr1* in pancreatic islets of parental C3H and NZO mice (B). Quantitative real-time PCR of *Kdelr*3 in NZO pancreatic islets treated with 500 µM palmitate and 25 mM glucose for 24h (Palm+Glu) (C). Data are presented as mean ± SEM and were analyzed by Students *t* test, *p < 0.05; **p < 0.01; ****p < 0.0001 n=5-8 (A, B), n=4-5 (C).

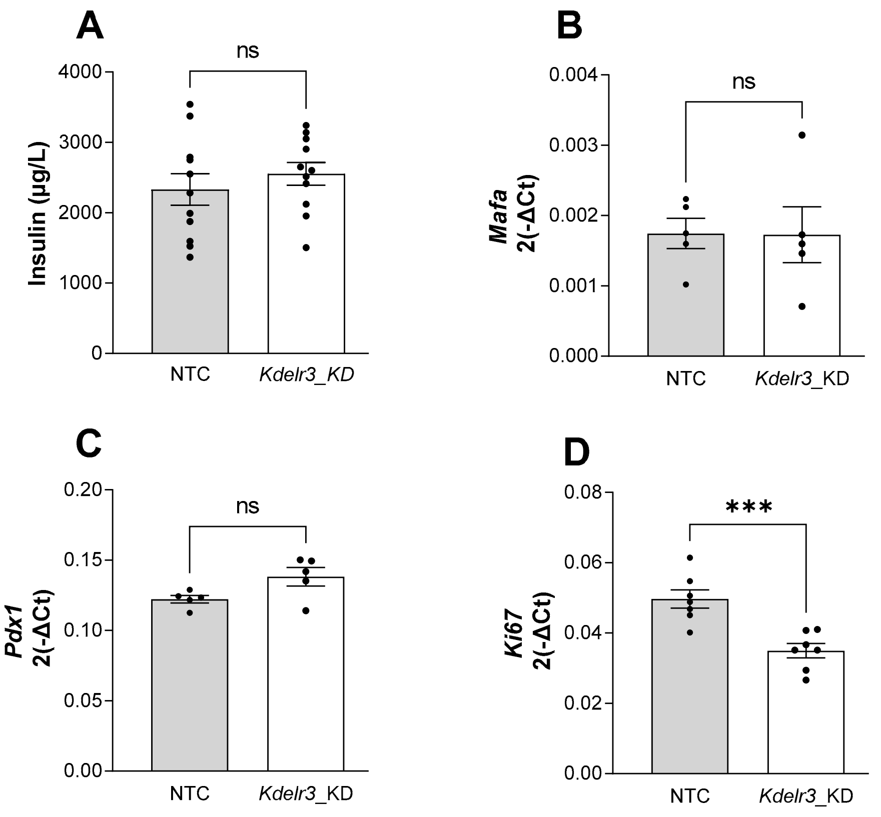

Supplemental Figure S3. Total insulin content in *Kdelr3*-KD MIN6 cells versus control (A), quantitative real-time PCR of *Mafa* (B), *Pdx1* (C) and *Ki67* (D) in *Kdelr3* knockdown MIN6 cells. *Kdelr3* knockdown cells were generated using a pool of siRNA. Data are presented as mean ± SEM and were analyzed by Students *t* test, *p < 0.05; **p < 0.01; ****p < 0.0001 n=5-10 independent experiments.

Supplemental Table S1: List of phenotyping and statistical testing of blood glucose development of *Nbg15* recombinant mouse strains.

The table indicates the mean, standard deviation and number of animals analysed. Statistical comparison used two-way ANOVA followed by Bonferroni post-hoc-test. The results reported by 95% confidence interval and adjusted P value refer to the comparison of the respective recombinant group (Prox.*Nbg15*$^{C3H/C3H}$, Dis.*Nbg15*$^{C3H/C3H}$, Con.*Nbg15*$^{C3H/C3H}$) with the control group (*Nbg15*$^{NZO/NZO}$).

Blood glucose development																		
	Nbg15$^{NZO/NZO}$			Prox.*Nbg15*$^{C3H/C3H}$					Dis.*Nbg15*$^{C3H/C3H}$					Con.*Nbg15*$^{C3H/C3H}$				
**Age (weeks)**	**Mean**	**SD**	**N**	**Mean**	**SD**	**N**	**95% CI of diff.**	**Adjusted P Value**	**Mean**	**SD**	**N**	**95% CI of diff.**	Adjusted P Value	Mean	SD	N	95% CI of diff.	Adjusted P Value
3	156.1	32.0	44	151.9	46.8	35	-65.21 to 56.65	>0.9999	172.8	27.9	35	-44.27 to 77.59	>0.9999	156.1	23.8	34	-61.45 to 61.41	>0.9999
4	216.0	28.0	31	214.2	33.5	20	-80.73 to 64.62	>0.9999	232.7	30.0	15	-63.89 to 92.31	>0.9999	225.1	27.1	22	-72.13 to 70.29	>0.9999
5	220.3	32.2	31	205.1	23.9	20	-94.13 to 51.23	>0.9999	244.9	32.5	15	-55.98 to 100.2	>0.9999	209.3	43.9	22	-92.24 to 50.18	>0.9999
6	238.4	73.7	38	205.6	40.7	25	-101.2 to 33.15	0.6751	202.0	60.8	24	-105.5 to 30.06	0.547	204.3	37.5	26	-98.60 to 34.73	0.753

7	252.0	91.4	31	221.4	81.1	20	-109.5 to 35.84	0.6736	248.1	36.0	15	-84.52 to 71.68	>0.9999	222.4	58.9	22	-110.9 to 31.53	0.5455
8	277.6	114.8	31	255.4	110.0	20	-101.1 to 44.29	>0.9999	289.9	95.6	15	-68.27 to 87.93	>0.9999	240.0	77.1	22	-118.9 to 23.52	0.3261
9	345.5	146.6	31	300.1	165.9	20	-124.2 to 21.12	0.2678	296.8	78.8	15	-129.3 to 26.93	0.3496	241.9	104.2	22	-184.8 to -42.39	0.0004
10	368.0	139.1	44	319.5	139.8	35	-109.4 to 12.42	0.1697	346.5	111.4	35	-82.41 to 39.45	>0.9999	255.4	112.8	34	-174.1 to -51.21	<0.0001
11	398.1	162.7	31	316.4	177.2	20	-160.7 to -15.31	0.0113	328.9	116.4	15	-149.8 to 6.385	0.0837	263.2	117.7	22	-216.2 to -73.80	<0.0001
12	417.8	170.9	31	312.7	161.4	20	-184.1 to -38.72	0.0007	331.5	121.2	15	-166.9 to -10.72	0.0195	264.2	117.2	22	-234.9 to -92.46	<0.0001
13	401.9	178.9	30	338.7	200.8	20	-144.7 to 1.310	0.0562	342.2	145.0	15	-142.9 to 13.95	0.1469	242.4	105.7	22	-243.3 to -100.2	<0.0001
14	393.7	184.0	29	338.3	187.6	20	-140.9 to 5.754	0.082	256.9	141.6	15	-224.0 to -66.55	<0.0001	235.9	123.8	22	-245.8 to -102.0	<0.0001
15	361.8	187.0	32	307.9	201.3	24	-135.5 to 3.138	0.0668	268.7	109.4	22	-171.3 to -29.94	0.002	237.8	122.1	26	-204.9 to -68.27	<0.0001

Supplemental Table S2. List of phenotyping and statistical testing of body weight development of *Nbg15* recombinant mouse strains.

The table indicates the mean, standard deviation and number of animals analysed. Statistical comparison used two-way ANOVA followed by Bonferroni post-hoc-test. The results reported by 95% confidence interval and adjusted P value refer to the comparison of the respective recombinant group (Prox.*Nbg15*$^{C3H/C3H}$, Dis.*Nbg15*$^{C3H/C3H}$, Con.*Nbg15*$^{C3H/C3H}$) with the control group (*Nbg15*$^{NZO/NZO}$).

Body Weight development																		
	Nbg15$^{NZO/NZO}$			Prox.*Nbg15*$^{C3H/C3H}$					Dis.*Nbg15*$^{C3H/C3H}$					Con.*Nbg15*$^{C3H/C3H}$				
Age (weeks)	Mean	SD	N	Mean	SD	N	95% CI of diff.	Adjusted P Value	Mean	SD	N	95% CI of diff.	Adjusted P Value	Mean	SD	N	95% CI of diff.	Adjusted P Value
3	13.2	0.4	44	15.1	0.6	35	-0.3117 to 4.062	0.1201	14.7	0.6	35	-0.6889 to 3.685	0.3024	14.0	0.3	34	-1.380 to 3.029	>0.9999
4	22.3	0.6	31	21.3	1.0	20	-3.160 to 2.050	>0.9999	20.1	0.9	15	-5.213 to 0.3834	0.1163	20.8	0.6	22	-3.993 to 1.114	0.5304
5	31.4	0.6	31	29.4	0.9	20	-4.214 to 0.9965	0.4171	30.3	0.8	15	-4.152 to 1.445	0.7393	30.4	0.5	22	-3.586 to 1.521	0.9974
6	38.0	0.5	38	36.2	0.5	25	-3.738 to 1.079	0.5579	37.8	0.8	24	-2.589 to 2.275	>0.9999	36.8	0.5	26	-3.514 to 1.269	0.7821
7	42.8	0.5	31	41.0	0.5	20	-4.062 to 1.148	0.5408	43.3	0.6	15	-2.575 to 3.021	>0.9999	42.2	0.7	22	-3.139 to 1.967	>0.9999
8	47.4	0.4	31	44.9	0.5	20	-4.629 to 0.5813	0.1884	49.1	0.4	15	-1.297 to 4.300	0.5961	45.9	0.7	22	-4.022 to 1.085	0.5045
9	49.9	0.4	31	47.8	0.3	20	-4.250 to 0.9602	0.391	51.8	0.5	15	-1.120 to 4.477	0.4523	49.3	0.8	22	-3.124 to 1.982	>0.9999
10	52.5	0.5	44	50.2	0.4	35	-4.456 to -0.08225	0.039	54.7	0.7	35	0.02069 to 4.395	0.047	53.0	0.7	34	-1.651 to 2.759	>0.9999
11	53.4	0.7	31	50.0	0.6	20	-5.594 to -0.3835	0.0181	58.0	0.6	15	1.528 to 7.125	0.0007	54.4	1.1	22	-1.527 to 3.580	>0.9999

12	54.4	0.8	31	51.3	0.8	20	-5.374 to -0.1632	0.0329	60.2	0.7	15	2.725 to 8.322	<0.0001	57.5	1.2	22	0.5909 to 5.697	0.0096
13	54.6	1.0	30	51.5	1.1	20	-5.408 to -0.1739	0.0321	60.9	1.1	15	3.159 to 8.777	<0.0001	58.3	1.2	22	1.031 to 6.160	0.0024
14	53.6	1.3	29	52.6	1.3	20	-3.279 to 1.979	>0.9999	60.3	1.1	15	3.625 to 9.266	<0.0001	60.0	1.2	22	3.830 to 8.985	<0.0001
15	54.6	1.2	32	53.0	1.3	24	-3.525 to 1.445	0.948	62.5	1.5	22	5.588 to 10.66	<0.0001	61.3	1.3	26	4.326 to 9.226	<0.0001

Supplemental Table S3. List of *Nbg15* scored genes according to variant effect predictor analysis.

Genes carrying potential disruptive mutations were subjectively scored by the variant effect predictor analysis (VEP_Ensembl) as "high" and "moderate". Highly impacting genes were scored by the presence of stop lost/gain predicted mutations. Furthermore, polymorphisms scored with moderate impact were further ranked using the Sorting Intolerant From Tolerant tool (SIFT- Open source tool) that predicts the impact of amino acid substitution according to sequence homology of the given amino acid exchange. Highly disruptive mutations were scored when SIFT <0.05. Considering a percentage of false positive/negative errors, all genes ranked by SIFT within the range of 0.05-0.5 were further considered for analysis. *gene not covered by microarray analysis in the analysed tissue. Sequence reference: **REL-1303 - GRCm38. SNP with low coverage (©).**

Chr.15 position (Mbp)	Gene symbol	dbSNP	C3H.HeJ	NZO.HlLtJ	Protein position	Amino acids	Codons	IMPACT	SIFT
71.806	*Col22a1**	rs46351738	C	T	1461	K/E	Aag/Gag	Moderate	0.00
63.825	*Gsdmc2*	rs252605414	C	G	407	L/V	Ctc/Gtc	Moderate	0.01
78.349	*Csf2rb*	rs4230845	C	A	742	S/R	Agt/Cgt	Moderate	0.01
74.540	*Adgrb1/Bai1*	rs51566550	A	G	379	R/H	cGc/cAc	Moderate	0.01
76.705	*Recql4*	rs215408973	G	A	967	A/V	gCt/gTt	Moderate	0.02
77.718	*Apol7e**	rs107983511	C	-	220	M/V	Atg/Gtg	Moderate	0.02
77.051	*Apol6*	rs261533897	G	C	122	E/D	gaG/gaC	Moderate	0.04
79.919	*Cbx7*	rs36397331	T	C	153	R/H	cGc/cAc	Moderate	0.05
52.040	*Aard*	rs31595794	T	A	103	T/S	Acg/Tcg	Moderate	0.07
76.421	*Mroh1*	rs214800774	G	C	404	D/H	Gat/Cat	Moderate	0.08
78.908	*Sh3bp1*	rs13463449	T	C	515	P/L	cCg/cTg	Moderate	0.08
79.525	*Kdelr3*	rs256912165	A	T	96	E/V	gAg/gTg	Moderate	0.08
77.389	*Apol7a*	rs229171902	G	A	272	T/I	aCc/aTc	Moderate	0.10
78.961	*Triobp*	rs31569891	A	G	168	T/A	Act/Gct	Moderate	0.10
78.938	*Nol12*	rs13466090	T	C	92	A/V	gCg/gTg	Moderate	0.11
71.464	*Fam135b*	rs49135881	C	T	485	V/I	Gtc/Atc	Moderate	0.17
75.896	*Eef1d*	rs227346636	C	A	155	K/N	aaG/aaT	Moderate	0.18
57.936	*Tbc1d31*	rs265189026	G	C	247	G/R	Ggg/Cgg	Moderate	0.22
79.828	*Cbx6*	rs227221465	C	G	309	Q/H	caG/caC	Moderate	0.22
76.888	*Zfp7*	rs31575067	T	G	72	E/D	gaG/gaT	Moderate	0.24
79.357	*Maff*	rs252068181	T	A	25	S/T	Tcg/Acg	Moderate	0.25
76.596	*Cpsf1**	rs248187700	G	A	1439	L/F	Ctc/Ttc	Moderate	0.26
57.251	*Slc22a22*	rs31794878	A	T	344	L/M	Ttg/Atg	Moderate	0.27
79.892	*Apobec3*	rs108537160	A	C	6	L/M	Ctg/Atg	Moderate	0.27
77.481	*Apol10a**	rs31475931	©	C	7	L/S	tTa/tCa	Moderate	0.34
66.542	*Tmem71*	rs256104481	A	T	214	D/V	gAt/gTt	Moderate	0.35
58.874	*Trmt12*	rs36461259	A	G	342	S/N	aGc/aAc	Moderate	0.36
76.870	*Zfp251*	rs47788016	A	G	78	L/F	Ctt/Ttt	Moderate	0.38

79.738	*Sun2*	rs232461033	T	C	165	R/G	Agg/Ggg	Moderate	0.38
75.712	*Rhpn1*	rs229443499	G	A	386	V/I	Gtc/Atc	Moderate	0.46
76.329	*Exosc4*	rs13465162	T	C	69	*/R	Tga/Cga	High	–
79.637	*Fam227a*	rs37552463	A	G	1	M/T	aTg/aCg	High	–
79.900	*Apobec3*	rs46309510	A	G	11	W/*	tgG/tgA	High	–
79.934	*Cbx7*	rs47269926	T	A	24	L/*	tTg/tAg	High	–

Supplemental Table S4. Chemicals and buffers.

Compound	Supplier
**<u>Anticoagulant coktail:</u>**	
**EDTA 0.5 M**	Sigma Aldrich, Steinheim, Germany
**Aprotinin 0.15 M**	Sigma Aldrich, Steinheim, Germany
**Heparin 10.000U/Ml**	Biochrom, Berlin, Germany
**Diprotin A**	Bachem, Bubendorf, Switzerland
Trizol	Qiagen, Crawley, U.K.
Chloroform (pure)	AppliChem, Darmstadt, Germany
Hexanucleotide primer	Roche, Mannheim, Germany
Insulin Actrapid® HM Penfill®	Novo Nordisk Pharma GmbH, Mainz, Germany
Isopropanol (≥ 99. 5 %)	AppliChem, Darmstadt, Germany
QIAzol® reagent	QIAGEN, Hilden, Germany
Sterile glucose 20 %	Braun (Melsungen, Germany)
Microvette CB 300 µL Lithium-Heparin	Sarstedt, Nümbrecht, Germany
PCR-Plates (FrameStar® 384-Well)	4titude, Berlin, Germany
Rotilabo microtest plates (96 well)	Carl Roth, Karlsruhe, Germany
Krebs-Ringer HEPES	15 mM HEPES, 5 mM KCl, 120 mM NaCl, 24 mM NaHCO3, 1 mM MgCl2, 2 mM CaCl2, and 1 mg/ml BSA
Lysis buffer	20 mmol/L Tris-HCl, 150 mmol/L sodium chloride, 1 mmol/L EGTA, 1 mmol/L EDTA, 1%(v/v) Triton-X-100 and a proteinase inhibitor and a phosphatase inhibitor cocktail

Supplemental Table S5. Genotyping primers for the recombinant congenic strains.

Mouse DNA was isolated from tail tips using the Invisorb® Genomic DNA Kit II (STRATEC Molecular GmbH, Berlin, Germany) according to manufacturer's instructions. For genotyping the PCR-based KASP (Kompetitive Allele-Specific PCR) method from LGC genomics (LGC group, Teddington, United Kingdom).

Primer	SNP ID	Position (Mbp)	C3HeB/FeJ	NZO/HlLtJ
15-005994001-M	rs3715343	6.12	C	A
15-033125499-M	rs3720676	32.96	G	A
15-041919640-N	rs3090057	41.56	C	T
15-049283927-M	rs3719583	48.89	A	C
15-059192855-M	rs3653368	58.6	G	A
15-063976267-N	rs3023419	63.34	T	A
15-071973707-N	rs3088506	71.35	G	T
15-074947367-M	rs3724474	74.31	T	A
15-080641980-N	rs4230879	79.74	A	G
15-087100507-M	rs3690689	86.13	G	A
15-093195380-M	rs3707104	92.14	T	C
15-098621793-N	rs3023427	97.54	T	C
15-102788257-N	rs3023429	101.64	T	G

Supplemental Table S6. Sequence information of *Kdelr3* siRNA pool of oligonucleotides.

Gene ID	Target sequence	Supplier
Kdelr3	1 GUAGUGUCUGGAGUUGUUC<br>2 CUGAGGUGCUAAAUAGAAU<br>3 GCACCAUCUUGGGAUAAAC<br>4 CUAAGAACCACCAAUGUAG	Dharmacon (Lafayette, CO, USA), #E-052192-00-0005, Accell Mouse Kdelr3 (105785) siRNA SMART pool

Supplemental Table S7. List of SYBR green primers for quantitative real-time PCR.

Primers were designed using primer designing tool from NCBI and synthesized by Eurogentec (Seraing, Belgium) and diluted at a final concentration of 10 nM. F: forward; R: reverse.

Gene symbol	Primer ID	Primer sequence (5 →3′)
Kdelr3	Kdelr3_F	GGCGGGCGGGACCAT
	Kdelr3_R	GAAAACCACCTTCATCACTGTGT
Kdelr1	Kdelr1_F	TTGACCTCATCGCCATCGTT
	Kdelr1_R	TTCCCCTTCAGGACTTTGGTG
Kdelr2	Kdelr2_F	TGGTGTCGTCCAGACCATTC
	Kdelr2_R	GCTTCTTCCCTTTGAGTACTTTTG
Ins2	Ins2-F	CCCTGCTGGCCCTGCTCTT
	Ins2-R	AGGTCTGAAGGTCACCTGCT
Cbx6	Cbx6 _F	GCCGAATCCATCATTAAACG
	Cbx6 _R	TTGGGTTTAGGTCCCCTCTT
Fam135b	Fam135b_F	TAGAGACCTGAGACAGCTCG
	Fam135b_R	TGATCTTTTTGGTTCATTACCTGTC
Znt8	Znt8_F	TGCCAAGTGGAGACTCTGTG
	Znt8_R	CTGCTCGATACCACCCAAAT
Pcsk1	Pcsk1_F	TCTGGTTGTCTGGACCTCTGAGT
	Pcsk1_R	CATCAAGCCTGCCCCATTCTTT
Pcsk2	Pcsk2_F	TCTGACTGTGCTCACCTCCAA
	Pcsk2_R	AGGACTCCGTAGCCAAAGAGG
Chop	Chop_F	CCACCACACCTGAAAGCAGAA
	Chop_R	AGGTGAAAGGCAGGGACTCA
Atf3	Atf3_F	CTCTGCGCTGGAGTCAGTT
	Atf3_R	GCCTCCTTTTCCTCTCAT
